# Investigating Unmet Health Needs in Primary Health Care Services in a Representative Sample of the Greek Population ^†^

**DOI:** 10.3390/ijerph10052017

**Published:** 2013-05-17

**Authors:** Evelina Pappa, Nick Kontodimopoulos, Angelos Papadopoulos, Yannis Tountas, Dimitris Niakas

**Affiliations:** 1 Faculty of Social Sciences, Hellenic Open University, Riga Fereou 169 & Tsamadou, Patras 26222, Greece; E-Mails: nkontodi@otenet.gr (N.K.); docpapado@yahoo.gr (A.P.); niakas@eap.gr (D.N.); 2 “ATTIKON” University Hospital, 1 Rimini Street, Athens 12462, Greece; 3 Centre for Health Services Research, Department of Hygiene and Epidemiology, Medical School, Athens University, 25 Alexandroupoleos Street, Athens 11527, Greece; E-Mail: chsr@med.uoa.gr

**Keywords:** unmet needs, health care system, equity, Greece

## Abstract

Unmet health care needs are determined as the difference between the services judged necessary and the services actually received, and stem from barriers related to accessibility, availability and acceptability. This study aims to examine the prevalence of unmet needs and to identify the socioeconomic and health status factors that are associated with unmet needs. A cross-sectional study was conducted in Greece in 2010 and involved data from 1,000 consenting subjects (>18 years old). Multiple binary logistic regression analysis was applied to investigate the predictors of unmet needs and to determine the relation between the socio-demographic characteristics and the accessibility, availability and acceptability barriers. Ninety nine participants (9.9%) reported unmet health needs during the 12 months prior to the research. The most frequently self-reported reasons were cost and lack of time. Youth, parenthood, physician consultations, and poor mental health increased the likelihood of unmet needs. Women were less likely to report accessibility and availability than acceptability barriers. Educational differences were evident and individuals with primary and secondary education were associated with significantly more accessibility and availability barriers compared with those with tertiary education. Unmet health needs pose a significant challenge to the health care system, especially given the difficult current financial situation in Greece. It is believed that unmet health needs will continue to increase, which will widen inequalities in health and health care access.

## 1. Introduction

Access to health care is a fundamental determinant of health and its equitable distribution across the population is a critical issue of health services research. The main target of health systems is to ensure equity in access to needed health care irrespective of socioeconomic status and other non-need characteristics. As Andersen points out [1], “equity in access to health care is best considered in the context of whether people in need of medical care receive it or not”.

Access to health care is systematically evaluated based on the common needed-adjusted model, according to which it is determined whether non-need factors affect the use of health services, after controlling for health need variables. Some of the limitations that have been expressed regarding this model were related to the lack of information provided for the non-users and to the fact that dimensions like adequacy, quality and appropriateness of the received care could not be included [2,3]. 

Unmet needs, which are defined as “the difference between services judged necessary to deal appropriately with health problems and services actually received” [4], are considered as simple tools in monitoring the accessibility and the extent of inequity in access and use of health care [5]. Past research has shown that unmet health needs result from barriers that are related to the health system and to characteristics or personal attitudes of the individuals [2,5,6,7]. According to these studies, the barriers were gathered into three categories including accessibility (related to cost and proximity), availability (related to the timely provision of health service), and acceptability (related to personal attitudes and circumstances). 

The difference between the services judged necessary and the services actually received imply a failure for people to improve their health status. In the European literature, the study of unmet needs as a determinant of access to healthcare is limited. Previous studies have shown that unmet needs may worsen health status and quality of life [8], increase the risk of mortality [9], or be related to symptoms of mental and psychosomatic nature [10]. Furthermore, it was found that the factors that put people at risk of having unmet needs were youth and old age, female gender, lack of insurance coverage, high educational level, low income or unemployment, and poor health status [2,11,12,13,14,15,16,17], implying an inequitable access to health care according to socio-economic status. 

In Greece, access to health services has been investigated based on the conventional need-adjusted utilization model [18,19,20,21] and the use of unmet health care needs, as an indicator of equitable access to health services, has been studied in the context of two European surveys [22,23]. The current study is the first single survey in Greece which attempts to investigate access to health care with respect to unmet needs. The objectives of the study were: (i) to estimate the prevalence of unmet health needs in Greece, (ii) to identify the factors that put individuals at a risk of having unmet health needs, and (iii) to investigate whether socio-demographic factors were associated with the three indicators of unmet health needs.

## 2. Methods

### 2.1. Study Design

The cross-sectional study was conducted in October 2010 and involved a national representative sample of adults aged 18 and over residing in urban and rural areas in Greece. The response rate was 66.6%, with 1,000 out of 1,500 consenting subjects agreeing to be interviewed face-to-face. Non-fluent Greek speakers and institutionalized subjects were excluded. Participants were grouped, in proportion to the Greek population, by socio-demographic characteristics, according to a three-staged sampling methodology. In the first stage, a random sample of building blocks was selected in proportion to size based on the 2001 Population Census of the National Statistical Service of Greece. In the second stage, in each selected area of blocks, households were randomly selected by systematic sampling and in the third stage an eligible participant was selected by simple random sampling in each household. 

### 2.2. Measurements

Unmet health needs, which represent the dependent variable, were measured based on the following single-item question: “During the past 12 months, was there ever a time that you felt you needed medical help (examination or treatment) but you did not receive it?” The response to the question was binary “yes/no” and the participants who reported that they had an unmet need were further asked to report the reasons (up to two) from the following choices: “could not afford to pay”, “did not know if the social security fund covered” and “distance”, representing the accessibility indicator, “professional help not available at the time required” and “waiting too long”, representing the availability indicator, and “did not know where to get help”, “did not trust doctors/services”, “too busy”, “negligence” and “other”, representing the acceptability indicator. 

The control variables, including socio-demographic and health status, were selected on the basis of previous studies related to healthcare utilization. Demographics included gender, age, marital status and existence of children. Education, according to three levels, (primary, secondary and tertiary) and employment (economically active such as employers and employees *vs*. inactive such as retired, housewives, students) were used as socioeconomic status indicators. Participants also reported their place of residence (urban/rural). 

Health status was proxied by self-assessed health, the existence of chronic diseases, and physician consultations during the previous 12 months. Self-assessed health was measured by the Greek version of the Short Form-12 Health Survey (SF-12) questionnaire, which has been validated in a national representative general population sample [24]. It was used to measure, on a scale of 0–100, the physical and mental health of the respondents, with higher scores reflecting better perceived physical and mental health. Morbidity from chronic diseases was based on the presence of at least one of thirteen chronic conditions (diabetes mellitus type I & II, hypertension, dyslipidemia, heart failure, coronary ischemic disease, irritable bowel syndrome, chronic bronchitis, asthma, osteoarthritis, Alzheimer’s, depression, and anxiety disorders). Finally, participants were asked to report any physician consultations during the previous twelve months (*i.e.*, any use contrasted with no use).

### 2.3. Statistical Analysis

Cross-tabulation was used to estimate the prevalence of the unmet needs categories and the specific reasons, and chi-square test to assess the differences of reporting unmet needs. The binary logistic regression model using forward Wald selection was applied in order to: (i) estimate the adjusted odds ratio (O.R.) and the 95% confidence intervals of the predictors of the unmet health care needs, controlling for socio-demographic and health status factors, and (ii) determine the association among the participants’ socio-demographic characteristics and the three categories of unmet health care needs. The results were considered statistically significant when *p* < 0.05 and all analyses were performed using SPSS v17.0.

## 3. Results

Table 1 presents the overall sample distribution, according to socio-demographic characteristics, and the respective portions of respondents reporting self-perceived unmet health care needs. Ninety nine out of 1,000 participants (9.9% of the sample) reported that they had not received a healthcare service that they needed during the 12 months prior to the study. Unmet needs were more common and significantly higher among females (gender differences remained consistent in terms of significance when investigating the three category indicators separately), those married and having children, those economically inactive, and those with poor health status, *i.e*., those having chronic diseases and consulting physicians. 

Table 2 presents the percentage contribution of each of the reasons for unmet health care needs. The two most common reasons for not seeking medical care were cost (25.4%) and being too busy, mainly due to family and work reasons (23.9%). Other reasons commonly stated were waiting lists (12.7%) and “didn’t know where to get help” (8.2%). The analysis also revealed that the largest barrier—accounting for 47.8% of the unmet needs overall—was acceptability, with problems stemming from personal circumstances and attitudes. Availability problems accounted for 18.6% and unmet needs attributed to accessibility problems were rated at 33.6%. About 20.2% reported a second reason different from the first category. Acceptability is the most common category selected as second choice.

Table 3 presents the results of the multiple binary logistic regressions of the unmet health care needs. Only the independent variables, socio-demographic and health status factors that contributed significantly to the model were controlled for. People in the 25–34 age group had more than two times higher likelihood of having unmet health care needs (O.R. 2.23) than elderly people (65+ years old). The existence of children is related to unmet health care needs and individuals with children were 44% more likely (O.R. 1.44) to report unmet needs compared with individuals with no children. Concerning health status proxies, it was observed that individuals who had physician consultations at least once were two times (O.R. 2.00) more likely to report unmet needs compared with those who had no physician consultations. Better mental health was associated with a lower likelihood of having unmet health care needs (O.R. 0.92), with each unit improvement in mental health resulting in an 8% reduction in the likelihood of reporting unmet health care needs. 

**Table 1:** Descriptive characteristics of the study population and those who reported unmet needs.

Variable	Study population (%)	Reported unmet needs (%)
**Total**		**9.9**
**Sex**		
Men	50.6	35.4
Women	49.4	64.6 *
**Age**		
18–24	8.6	6.1
25–34	19.8	19.2
35–44	19.0	20.2
45–54	18.2	12.1
55–64	14.8	16.1
65+	19.6	26.3
**Marital Status**		
Single	28.5	17.2
Married	58.7	61.1
Divorced/Widowed	12.8	21.2 **
**Children**		
Yes	65.3	77.8
No	34.7	22.2 *
**Education**		
Primary	22.2	28.3
Secondary	58.6	55.6
University	19.1	16.2
**Occupation**		
Employer	19.0	19.2
Employee	30.5	18.2
Retired	22.5	31.3
Other	27.8	31.3 **
**Urbanity**		
Urban	72.1	70.7
Rural	27.9	29.3
**Physician Consultations**		
Yes	66.7	83.8
No	33.3	16.2 *
**Chronic Diseases**		
Yes	41.0	57.9
No	59.0	42.1 *

Note: * *p* < 0.0001; ** *p* < 0.001 according to chi-square test.

**Table 2:** Distribution of reasons for unmet health needs.

	%
**Accessibility**	**33.6**
Cost	25.4
Social security coverage	5.2
Distance	3.0
**Availability**	**18.6**
Waiting too long	12.7
Professional help not available when required	6.0
**Acceptability**	**47.8**
Too busy	23.9
Didn’t know where to get help	8.2
Didn’t trust doctors/services	4.5
Negligence	4.5
Other	6.7
Accessibility + Availability	2.0
Accessibility + Acceptability	8.1
Availability + Acceptability	10.1

**Table 3:** Adjusted odds ratio (O.R.) of unmet needs according to various socio-demographic characteristics.

Factors	O. R.	sig.	C.I. 95%
Sex (men)			
Women	1.27	NS	0.79–2.04
Age (65+)			
18–24	1.57	NS	0.52–4.73
25–34	2.23	*p* < 0.05	1.02–4.86
35–44	1.61	NS	0.80–3.26
45–54	0.63	NS	0.29–1.39
55–64	1.02	NS	0.49–2.11
Children (no)			
Yes	1.44	*p* < 0.001	1.11–1.85
Physician consultations (no)			
Yes	2.00	*p* < 0.05	1.11–3.64
PCS-12	1.006	NS	0.98–1.04
MCS-12	0.92	*p* < 0.0001	0.90–0.94

For categorical variables the reference group is indicated into the parenthesis; PCS-12 = Physical Component Score, MCS-12 = Mental Component Score; NS = Non Significant (*p* < 0.05); C.I. = Confidence Intervals.

Table 4 shows the results of the association between selected socio-demographic characteristics and the indicators of the unmet needs. The participants who reported two reasons were categorized according to the first one, because it was assumed that the first reason is of higher priority. Because the availability group was small, it was merged with the accessibility group as barriers related more to health system characteristics and the remaining acceptability group acted as the reference category. Women were approximately 80% less likely (O.R. 0.19) to report accessibility/availability barriers rather than acceptability barriers. Education proved to be a very important determinant, with people with primary or secondary education being above six (O.R. 6.10) and seven (O.R. 7.51) times more likely, respectively, to report accessibility and availability than acceptability barriers compared with tertiary level people. 

**Table 4:** Adjusted odds ratio (O.R.) relating the three indicators of unmet needs to selected socio-demographic characteristics.

Factors	O. R.	sig.	C.I. 95%
Sex (men)			
Women	0.19	*p* < 0.001	0.06–0.60
Education (university)			
Primary	6.10	*p* < 0.05	1.32–28.19
Secondary	7.51	*p* < 0.001	1.85–30.51
Occupation (active)			
Inactive	2.46	NS	0.85–7.13

For categorical variables the reference group is indicated into the parenthesis. NS= Non Significant (*p* < 0.05). C.I. = Confidence Intervals.

## 4. Discussion

The present study aimed to shed light on the unmet health care needs of the Greek population by investigating the distribution of reasons for which self-assessed needed health care is not received. Our findings indicate that socio-demographic characteristics and health status are important factors in explaining the unmet health care needs. 

About 10% of the respondents reported unmet needs. This rate is significantly increased compared with the findings of the international European Union-Survey on Income and Living Conditions (EU SILC) [23] carried out in 2004 in 14 European countries. In the six-year period between 2004 and 2010, self-assessed unmet needs for health care almost doubled from 5.26% to 9.9%. Furthermore, another study has shown a significant increase of 15% in unmet health needs during a two-year period [25]. 

The most common reason was cost, and the obvious explanation concerns the health system’s characteristics. The Greek health care system is a mixed public-private one in terms of organization, financing and provision [18]. Primary health care is poorly organized and highly fragmented. The attempt in 2012 to merge the four biggest insurance funds into one (EOPYY-National Organization for Health Care) has not yet solved the organizational, financial and provision issues. Greek people have faced unequal access to standard services due to entitlement, geography and ability to pay [19]. The gap in the inconsistencies of the public health sector is covered by the overdeveloped private sector and people’s ability to pay. The second most common reason is “too busy,” which means that unmet needs must be understood within a wider social context. Indeed, the responsibilities of family and work affect individuals’ ability to access health care and this is explained by the results of our study below, where the existence of children is a significant determinant. 

Interesting differences were found in unmet needs by age even though disparities were not statistically significant among all age groups. Young age was associated with more unmet health care needs, a finding that has been identified in previous studies [1,26,27]. Possible explanations could be related to perceptions of health care benefits or the quality of care. A recent study in Greece [28] has shown that age is a significant determinant of patients’ perceptions of primary health care quality. According to this, people have a different understanding of health care which in turn influences their perception of the quality of services. Another possible explanation is young people’s lack of knowledge about health care resources, different assessment of symptoms, and young adults’ inappropriate use of health care [26] or the fact that younger patients report least satisfaction with health care [29].

The association between health care use and unmet needs for health care was somewhat surprising. Our results revealed that physician consultations increase the likelihood of reporting unmet needs by about two times, a finding consistent with previous studies [14,26]. It seems that a significant parameter in this association is the adequacy of the received care. Information about the quality, quantity and the timelines of the care received is very important in order to explain the above association. As Allin *et al*. [3] explain, if a service is not received on time, an individual perceives an unmet need and still requires further care because his/her health status has not improved and finally reports unmet needs as the care was inadequate. From another perspective, health care users are more aware of the limitations and shortcomings of the health care delivery system, and probably have more needs that have not been met properly because the received care was inadequate, resulting in unfulfilled expectations and dissatisfaction. 

Poor health status and mainly self-assessed mental health was related to higher unmet health needs. Unmet needs for mental health care are well documented and psychosocial reasons are significant for not seeking needed mental care. There is evidence that access to mental health care is limited due to stigma and discrimination [30] or the fear of treatment [31,32]. 

Studying the socio-demographic factors associated with accessibility, availability and acceptability barriers highlighted the importance of female gender as a determinant of unmet needs stemming from personal circumstances. Women reported more acceptability barriers in asking for health care. Female gender, as a significant predictor of unmet needs, is common and its unique role is related to women’s double role and their responsibilities in the workplace and at home. Previous results [33] have shown that working outside home and providing unpaid care to their families may impede the ability of women to seek care for themselves. This is justified by our results, where the existence of children increases the likelihood of reporting unmet health needs by about 44%. 

Furthermore, our results have identified educational disparities in unmet health needs. People in the lower and middle educational levels (representing the low and middle social classes) reported huge accessibility and availability barriers in comparison to those with university education. The different utilization patterns according to educational level could be an explanation. It has been found in a previous study that people with higher education use more private primary health services, paying out-of-pocket for needed health services [18] in order to fulfill, among other things, their great expectations. On the contrary, primary and middle-level educated people use more public primary services, facing the inconsistencies of the public health sector that were mentioned before. Considering further that low education is associated with low-qualified and low-paying jobs, there is evidence that income-related inequalities may exist since low income is associated with accessibility barriers [13,21,34] and the more vulnerable may not receive the needed care due to cost. 

Our results should be interpreted in the light of some limitations. First of all, even though it is a cross-sectional study, the one year duration is subject to recall biases. Secondly, the size of those reporting unmet needs is small (in frequency terms), although in proportional terms it corresponds to 10% of the national representative Greek sample and it is totally comparable. This implies the possibility of under/over estimation, and it is difficult to assess the potential effect of this. Even though studies based on interviews are costly, further study with a bigger sample size is needed. Thirdly, the investigation of unmet needs is based on a single-item question which affects the measurement and the interpretation of the results. Monitoring unmet needs as a homogenous variable may underestimate the real unmet needs and mislead the policy implications. Different types of health services have different utilization patterns and different equity implications. It is very important to know the unmet needs for each type of health services (family doctor, specialist, and medication) and furthermore to relate them to specific reasons. On the other hand, it has been stated that the measurement of unmet needs using a set of questions rather than a single-item question could clarify whether the reported unmet needs are based on expectations or on clinical needs [35]. 

Future research considering the above limitations is needed to provide more insight into equity implications of unmet health care needs. Further information on the quality, quantity and the type of health services could be helpful to investigate in depth the variety of factors associated with unmet health care needs. 

## 5. Conclusions

This is the first national study conducted to measure the unmet health care needs of the adult population in Greece. Despite the above mentioned limitations, the results suggest that unmet health care needs may complement the assessment of access to health care. Unmet needs were related both to individuals and health system characteristics and important educational differences were raised. Attention should be paid by health care policy makers and focused policy actions should be undertaken in order to facilitate appropriate help-seeking and service use. Unmet health needs pose a significant challenge to the health care system. The current financial situation in Greece is very difficult, and it is believed that unmet health needs will continue to increase, widening at the same time inequalities in health and health care access. 
